# Markov-Modulated Continuous-Time Markov Chains to Identify Site- and Branch-Specific Evolutionary Variation in BEAST

**DOI:** 10.1093/sysbio/syaa037

**Published:** 2020-05-16

**Authors:** Guy Baele, Mandev S Gill, Paul Bastide, Philippe Lemey, Marc A Suchard

**Affiliations:** 1 Department of Microbiology, Immunology and Transplantation, Rega Institute, KU Leuven, Herestraat 49, 3000 Leuven, Belgium; 2 Department of Biostatistics, Jonathan and Karin Fielding School of Public Health, University of California, Los Angeles, CA 90095, USA; 3 Department of Biomathematics, David Geffen School of Medicine at UCLA, University of California, Los Angeles, CA 90095, USA; 4 Department of Human Genetics, David Geffen School of Medicine at UCLA, Universtiy of California, Los Angeles, CA 90095, USA

## Abstract

Markov models of character substitution on phylogenies form the foundation of phylogenetic inference frameworks. Early models made the simplifying assumption that the substitution process is homogeneous over time and across sites in the molecular sequence alignment. While standard practice adopts extensions that accommodate heterogeneity of substitution rates across sites, heterogeneity in the process over time in a site-specific manner remains frequently overlooked. This is problematic, as evolutionary processes that act at the molecular level are highly variable, subjecting different sites to different selective constraints over time, impacting their substitution behavior. We propose incorporating time variability through Markov-modulated models (MMMs), which extend covarion-like models and allow the substitution process (including relative character exchange rates as well as the overall substitution rate) at individual sites to vary across lineages. We implement a general MMM framework in BEAST, a popular Bayesian phylogenetic inference software package, allowing researchers to compose a wide range of MMMs through flexible XML specification. Using examples from bacterial, viral, and plastid genome evolution, we show that MMMs impact phylogenetic tree estimation and can substantially improve model fit compared to standard substitution models. Through simulations, we show that marginal likelihood estimation accurately identifies the generative model and does not systematically prefer the more parameter-rich MMMs. To mitigate the increased computational demands associated with MMMs, our implementation exploits recent developments in BEAGLE, a high-performance computational library for phylogenetic inference. [Bayesian inference; BEAGLE; BEAST; covarion, heterotachy; Markov-modulated models; phylogenetics.]

Molecular sequence evolution is typically modeled by Markov models of character substitution acting along the branches of a phylogenetic tree. These models are phenomenological descriptions of the evolution of DNA as a string of a number of discrete character states, with models of nucleotide substitution among four states being the most widely used in statistical phylogenetics. The Markovian property within such a model reflects the common assumption that evolution has no memory. Further, it is standard to assume that the Markov model is time-homogeneous, so that it can be characterized by a generator or instantaneous rate matrix $\mathbf{Q}_{}$ that remains constant during evolution (Gascuel and Guindon 2007). Early probabilistic phylogenetic reconstruction methods assumed a single substitution model that acted independently across all sites and lineages.

The characters at different alignment sites, however, typically evolve under varying structural or functional constraints, inspiring models that accommodate among-site rate variation by scaling up or down the expected number of substitutions at different sites. Sites evolve, nonetheless, in more qualitatively different ways than simply variation in their overall substitution rates (Pagel and Meade 2004). Furthermore, selective pressures vary over time and often defy *a priori* site partitioning into sets with approximately equal selection across an alignment. Examples of such a complex interplay between sites come from studies on how the 3D structure of proteins evolves over time. These studies show that, although a few essential sites may be invariable over long periods of evolutionary time, most sites do change their functional environment—and as a result, the functional constraints they are subjected to—during evolution (Penny et al. 2001). In order to capture and accurately model these types of evolutionary phenomena, there is need for a class of flexible substitution models that do not require prior knowledge regarding data partitioning.

The increase in computational power over the past two decades has enabled fast evaluation of complex models in a feasible amount of time, by focusing on exploiting many-core computing solutions (Suchard and Rambaut 2009). This has paved the way for evaluating high-dimensional substitution models and modeling complex scenarios, such as clade-specific and even branch-specific evolutionary processes. Markov-modulated models (MMMs) constitute a class of mixture models that allow the substitution process to change across each branch and this for each site independently within an alignment (we refer interested readers to Supplementary materials available on Dryad at https://doi.org/10.5061/dryad.230s5h0 for an in-depth introduction). In this article, we introduce a Bayesian inference framework for MMMs, with an implementation in BEAST (Suchard et al. 2018)—a software package for Bayesian evolutionary analysis—that accommodates phylogenetic uncertainty. In doing so, we strive for optimal generality by allowing switching between evolutionary models within the MMM that have different substitution rates, relative character exchange rates and stationary distributions.

## Methods

### Markov-Modulated Model Structure

Consider an MMM composed of $K$ evolutionary models (irrespective of those models being nucleotide, amino acid, or codon models). Each evolutionary model is defined by a relative substitution rate multiplier $\rho_{k}$ and a substitution model characterized by an instantaneous rate matrix $\mathbf{Q}_{k} = \left\{ Q^{(k)}_{s s} \right\}$, of dimension $S \times S$, and stationary distribution $\boldsymbol{\Pi}_{k} = \left( \pi_{k 1}, \ldots, \pi_{k S} \right)$. We also adopt the usual constraint -$\sum_{s = 1}^{S} Q^{(k)}_{s s} \pi_{k s} = 1$. The switching process between the $K$ models is defined by a $K$-state continuous-time Markov process with rate matrix

(1.1) \begin{equation*} \label{eq:switching} \mathbf{\Phi} = \left( \begin{array}{ccccc} -\sum_{k~~/{\hspace{-2.1mm}} =~ 1}\phi_{1k} & \phi_{12} & \cdots & \phi_{1K} \\ \phi_{21} & -\sum_{k~~/{\hspace{-2.1mm}} =~~ 2}\phi_{2k} & & \vdots \\ \vdots & & \ddots & \phi_{K -1,K} \\ \phi_{K 1} & \cdots & \phi_{K, K -1} & -\sum_{k~~/{\hspace{-2.1mm}} =~ K}\phi_{K k} \end{array} \right), \end{equation*}

where the element $\phi_{ij}$ corresponds to the rate of switching from substitution model $i$ to substitution model $j$, and the diagonal elements are fixed such that the rows sum to $0$. We denote the stationary distribution of this switching process by $\boldsymbol{\Psi} = \left(\psi_1, \ldots, \psi_K\right)$. These model switches follow a homogeneous, stationary—but not necessarily time-reversible—Markovian process. In Equation 1.1, we do not make use of an additional parameter $\delta$ that expresses the global rate of change between the evolutionary models because this is a deterministic parameter obtained by normalizing the model-switching process (Guindon et al. 2004; Gascuel and Guindon 2007).

The MMM is characterized by a $KS \times KS$ rate matrix $\mathbf{\Lambda}$ (Fischer and Meier-Hellstern 1993):
(1.2)
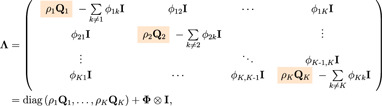

where $\mathbf{I}_{}$ is an $S \times S$ identity matrix and $\otimes$ denotes the Kronecker product. The MMM can therefore be considered a single Markov process with a state space equal to the Cartesian product of the state space of the switching process (between the evolutionary models) and the state space of the evolutionary models, with cardinality $K S$ and stationary distribution $\boldsymbol{\Pi}_{\mathbf{\Lambda}} = \left( \psi_{1}\pi_{11}, \ldots, \psi_{1}\pi_{1S}, \ldots, \psi_{K}\pi_{K1}, \ldots, \psi_{K}\pi_{KS} \right)$ (Guindon et al. 2004). As noted by Gascuel and Guindon (2007), the MMM in Equation 2 allows for every compound state $(k, s)$ to either: 1) stay in model $k$ and transition to $(k, s^{\prime})$ with rate defined by $\rho_{k} \mathbf{Q}_{k}$, or 2) change evolutionary models and transition to $(k^{\prime}, s)$ with rate $\phi_{k k^{\prime}}$. All rows in $\mathbf{\Lambda}$ sum to $0$, and because $\boldsymbol{\Psi}\mathbf{\Phi} = 0$ and $\boldsymbol{\Pi}_{k}\mathbf{Q}_{k} = 0$, it follows that $\boldsymbol{\Pi}_{\mathbf{\Lambda}}\mathbf{\Lambda} = 0$. We refer to Supplementary material available on Dryad for additional information on these MMMs, for example on their identifiability when combining them with among-site rate variation (ASRV; Yang 1994, 1996).

### Likelihood

In this section, we adopt a similar notation to Gascuel and Guindon (2007) to describe the data likelihood under an MMM. Likelihood calculations for MMMs employ a standard pruning approach (Felsenstein 1981), with integration over the compound states (i.e., the evolutionary model and character state) at the internal nodes of the tree, and integration over the unobserved categories at the tips. Let $\mathbf{Y} = (\mathbf{Y}_1, \ldots, \mathbf{Y}_{L}),$ where $\mathbf{Y}_{\ell}$ are the extant characters observed at aligned site $\ell$ for $\ell = 1, \ldots, L$, and let ${\cal T}$ denote the phylogenetic tree with its branch lengths. Let ${\cal M(\boldsymbol{\theta}, \boldsymbol{\phi})}$ denote the MMM that models the evolutionary process for all sites, where $\boldsymbol{\theta} = \{\boldsymbol \theta_1, \ldots, \boldsymbol \theta_{K}\}$ and $\boldsymbol\theta_k$ represents parameters for the $k$th evolutionary model, and $\boldsymbol{\phi}$ parameters of the switching process. The observed data likelihood is:

(1.3) \begin{equation*} L(\boldsymbol{\theta}, \boldsymbol{\phi}, {\cal T}, {\cal M} \mid \mathbf{Y}) = \prod_i \left( \sum_{(k, s)} \psi_{k}\pi_s L_i^R((k,s), \boldsymbol{\theta}, \boldsymbol{\phi}, {\cal T}, {\cal M} \mid \mathbf{Y}_i) \right), \end{equation*}

where the product is taken over every site $i$ in the alignment, with each site assumed to evolve independently. The sum over the compound states $(k, s)$ replaces the sum over the nucleotide characters $s$ that is performed for standard nucleotide substitution models (Gascuel and Guindon 2007). Here, $L_i^R((k,s), \boldsymbol{\theta}, \boldsymbol{\phi}, {\cal T}, {\cal M} \mid \mathbf{Y_i})$ is the likelihood of the data at site $i$ under category $k$ and given that state $s$ is observed at site $i$ of the root node $R$. We can generalize this notation as $L_i^v((k,s), \boldsymbol{\theta}, \boldsymbol{\phi}, {\cal T}, {\cal M} \mid \mathbf{Y_i})$ for node $v$ to express the partial likelihood of observing the characters at site $i$ in the extant sequences descending from $v$. This notation can be shortened to $L_i^v(k,s)$ because $\boldsymbol{\theta}$, $\boldsymbol{\phi}$, ${\cal T}$, ${\cal M,}$ and $\mathbf{Y}$ are the same for all sites and nodes. Let $l$ and $r$ be the left and right descendants of $v$ and $t_{v}$ the length of the branch connecting $v$ to its parent. Each partial likelihood is then defined as follows (taking into account that the evolutionary categories are unobserved; Gascuel and Guindon 2007):

(1.4) \begin{equation*}\begin{aligned} L_i^v(k, s) = \left \{ \begin{array}{l} \textrm{1 if } v \textrm{ is a leaf with nucleotide character} s, \\ \textrm{0 if } v \textrm{ is a leaf with nucleotide character}\\ \textrm{different from } s \textrm{ or} \\ \left( \sum_{(k', s')} P_{(k,s)(k',s')}(t_{l})L_i^l(k', s') \right) \left( \sum_{(k', s')}\right.\\ \left.P_{(k,s)(k',s')}(t_{r})L_i^r(k', s') \right) \textrm{ otherwise}. \end{array} \right.\ \end{aligned}\end{equation*}

The substitution probabilities $P_{(k,s)(k',s')}(t)$ are computed using matrix exponentiation of $\mathbf{\Lambda}$ with computational complexity ${\cal O} \left( K^3 S^3 \right)$ (Pan and Chen 1999), although lower complexity may be achieved depending on the Kronecker structure of $\boldsymbol{\Lambda}$ (but see the Supplementary material available on Dryad). Computing these probabilities for all ${\cal O} \left( N \right)$ branches in the phylogeny therefore sports a complexity of ${\cal O} \left( N K^3 S^3 \right)$. Evaluating the $L$ site likelihoods through the tree-pruning (or peeling) algorithm (Felsenstein 1981) amounts to a complexity of ${\cal O} \left( N L K^2 S^2 \right)$. Taken together, with a relatively small cost ${\cal O} \left( L \right)$ for taking logarithm of site likelihoods and summing over sites results in a computational complexity of ${\cal O} \left( N K^3 S^3 + N L K^2 S^2 \right)$ for the log-likelihood of the observed data.

### Implementation

We have implemented MMMs and their corresponding likelihood function in BEAST (Suchard et al. 2018), a widely used software package for Bayesian phylogenetic and phylodynamic inference using Markov chain Monte Carlo integration. These models are available for use in BEAST through XML specification, allowing to construct a wide range of different modeling assumptions such as the ones detailed in this article (and the Supplementary material available on Dryad). The use of MMMs substantially increases computation time in likelihood-based inference, and we offload the computationally demanding aspects to powerful multi- and many-core hardware through the BEAGLE library (Ayres et al. 2019).

## Biological Examples

We here consider substitution models that are time-reversible and therefore substitution model $k$ will have instantaneous rates $Q^{(k)}_{ij}$ that can be expressed in terms of base frequencies $\pi_{kj}$ and symmetric rate parameters $R^{(k)}_{i \leftrightarrow j} = R^{(k)}_{j \leftrightarrow i}$ as follows:

(1.5) \begin{equation*} Q^{(k)}_{ij} = \pi_{kj} R^{(k)}_{i \leftrightarrow j}. \end{equation*}

Thus a substitution model can be specified in terms of its base frequencies and symmetric rate parameters $\textbf R_k = \{R^{(k)}_{i \leftrightarrow j} | i ~~/{\hspace{-2.1mm}} =~ j, (i,j) \in \mathcal S \} $.

We adopt the following notation: MMM($M$)$_{ijkl}$, where $M$ denotes the type of substitution model and $i$, $j$, $k$, and $l$ denote the numbers of distinct sets of symmetric rate parameters, sets of base frequencies, the relative rate multipliers, and the structure of $\mathbf{\Phi}$ as either symmetric/triangular ($T$) or asymmetric ($A$), respectively. For example, an MMM(HKY)$_{222T}$ refers to an MMM featuring two different HKY substitution models, each with its own set of symmetric rate parameters and set of base frequencies, two different relative rate multipliers and a symmetric rate switching matrix $\mathbf{\Phi}$. An MMM(HKY)$_{122A}$ refers to an MMM featuring two different HKY substitution models that share the same set of symmetric rate parameters but have different sets of base frequencies, along with two different relative rate multipliers and an asymmetric rate switching matrix $\mathbf{\Phi}$. When the relative rate multipliers are all fixed to 1 to superimpose an ASRV model (see Supplementary material available on Dryad), the $k$ subscript is omitted (e.g., MMM(HKY)$_{22A}$).

We here consider two empirical data sets that show the importance of employing MMMs to accurately model the substitution process, as supported by Bayesian model selection. In Supplementary material available on Dryad, we analyze two additional empirical data sets—a plant plastid gene and an influenza A virus data set—that provide evidence in favor of MMMs over traditional substitution models but also showcase the wide range of modeling assumptions possible within our MMM formulation.

### Bacterial 16S Ribosomal RNA

Differences in base composition throughout the genome can bias phylogenetic inference when not properly taken into account. Often, the proportion of A+T in a genome differs from that of G+C, and different organisms exhibit different patterns of base composition. At the level of the entire genome, GC content varies greatly within and among major groups of organisms, which can skew phylogenetic reconstruction if not properly unaccounted for (Mooers and Holmes 2000). Two different evolutionary processes have been singled out as possible explanations for varying patterns of base composition: biases in the underlying process of mutation, as similar levels of GC content are often found in regions with different functional constraints, and natural selection, with increased global GC content in bacteria possibly being selected for by UV exposure (Singer and Ames 1970).

Environmental variation shaping nucleotide composition may cause unrelated taxa to share similar base composition and therefore be grouped together within a clade. To accurately reconstruct evolutionary histories through phylogenetic inference, these potentially differing base compositions need to be accommodated in an explicit manner by the nucleotide substitution model. To address this, Blanquart and Lartillot (2006) developed a nonstationary and nonhomogeneous model accounting for compositional biases, allowing the composition to change at random points in the tree, with the total number of change points across the tree being inferred from the data. Through a Bayesian analysis of eubacterial 16S rRNA and BAS1 gene yeast data sets, the authors show that in most cases, the stationarity assumption was rejected in favor of their nonstationary model.

We evaluate our MMM framework on 16S ribosomal RNA of five bacterial sequences: *Deinococcus radiodurans*, *Thermus thermophilus*, *Thermotoga maritima*, *Aquifex pyrophilus,* and *Bacillus subtilis* (GenBank accession numbers: Y11332.1, AJ251939.1, NR_029163.1, M83548.2, and CP009796.1). We use standard nucleotide substitution models as well as MMMs to infer their evolutionary history while fixing the *Aquifex pyrophilus* sequence as an outgroup. Given that the data contain three thermophilic (high GC content) and two mesophilic (lower GC content) bacteria genera (Mooers and Holmes 2000), we consider only MMM($*$)$_{22*}$ models and do not further explore higher-dimensional models. The true tree topology of this eubacterial data set is believed to group *D. radiodurans* and *T. thermophilus* together to the exclusion of *B. subtilis*, *T. maritima*, and *A. pyrophilus*, given that *D. radiodurans* and *T. thermophilus* share the same peptidoglycan and menaquinone type (Murray 1992). However, phylogenetic reconstruction under stationary models has a tendency to erroneously group *D. radiodurans* and *B. subtilis* together, because these mesophiles have similar, relatively low GC content.

**Figure 1:**
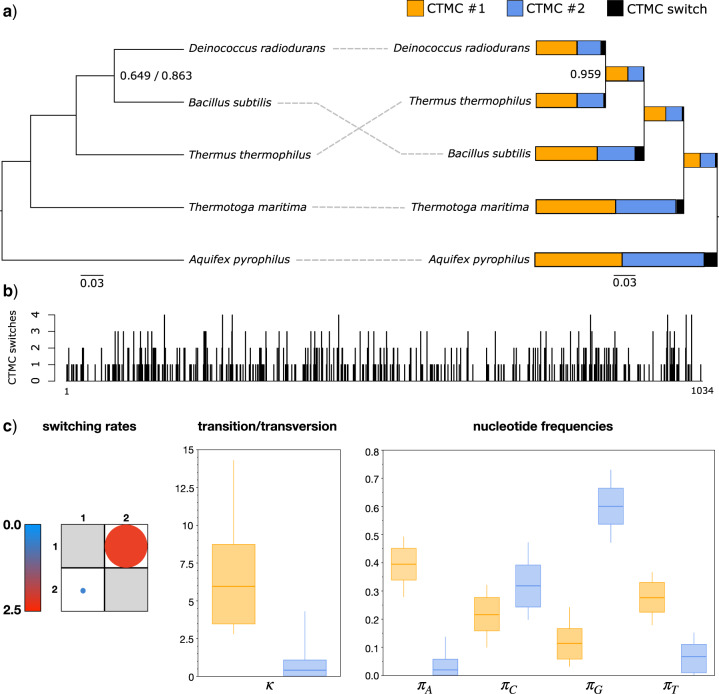
a) Maximum clade credibility (MCC) phylogeny relating five bacterial 16S sequences; unlabeled nodes have $>$0.9999 posterior probability. Standard nucleotide substitution models that assume among-site rate variation (ASRV) erroneously cluster the two mesophiles together with high posterior probability (0.649 for HKY and 0.863 for GTR in the topology on the left). However, an MMM(HKY)$_{22A}$ yields the correct clustering of the *Deinococcus radiodurans* and the *Thermus thermophilus* sequences with high posterior probability (topology on the right); each branch is annotated with the proportion of sites in each of the continuous-time Markov chain (CTMC) models, based on the maximum a posteriori (MAP) phylogeny. b) Number of CTMC model switches per alignment site based on the most probable hidden state realizations of the MMM on the MAP phylogeny; of the full alignment of 1304 sites, 761 sites are estimated not to switch between CTMC models. c) Mean posterior parameter estimates of the MMM show asymmetric switching between models (with circle sizes proportional to rate switching intensity) with pronounced differences in transition/transversion ratios and base frequencies.

Figure 1 shows the results of the phylogenetic reconstructions, with the HKY and GTR models—both featuring an ASRV model and a relaxed molecular clock with an underlying lognormal distribution—yielding similar (log) marginal likelihoods (we refer to Supplementary material available on Dryad for details on the marginal likelihood estimation procedure). Note that, because we will include an ASRV model in all of these MMMs, we set all $\rho_{i}$ in Equation 1.2 to 1 to ensure identifiability. Both the HKY and GTR models express strong support in favor of a clustering of *D. radiodurans* and *B. subtilis* (see Fig. 1), with the GTR model yielding a small increase in model fit to the data over the HKY model (log BF $<$ 1). As such, both models yield an incorrect clustering, which appears to be primarily based on both sequences being mesophilic (low GC content), whereas the three other sequences are considered thermophilic (high GC content). While an MMM of the type introduced by Tuffley and Steel (1998) offers no improvement over these models when $\mathbf{Q}_{1}$ is parameterized as an HKY model (see Supplementary material available on Dryad for the model’s details), a significant improvement in model fit can be obtained when $\mathbf{Q}_{1}$ is parameterized as a GTR model (log BF = 19). However, any MMM with two sets of base frequencies and with either a single set of symmetric rate parameters (an MMM($*$)$_{12*}$) or with two different sets of symmetric rate parameters (an MMM($*$)$_{22*}$) offers a further improvement in model fit compared to the standard nucleotide substitution models tested (8 $<$ log BF $<$ 45; we refer to Supplementary material available on Dryad for the log marginal likelihood estimates). This can be attributed to the fact that MMMs are able to accommodate differing base compositions throughout the tree topology, and consequently yield an accurate phylogenetic reconstruction of the bacterial relationships, with the *D. radiodurans* and *T. thermophilus* clustering together (see Fig. 1) (Embley et al. 1993; Mooers and Holmes 2000).

The base frequency estimates for the CTMC models within the MMM reflect the presence of mesophilic sequences (low GC content; orange in Fig. 1) and thermophilic sequences (high GC content; blue in Fig. 1) in our data. Despite the fact that only eight branches connect the observed sequences, alignment sites switch up to four times between CTMC models across the phylogeny, indicating evolutionary dynamics that cannot possibly be accommodated using standard nucleotide substitution models. Over 40% of the alignment sites undergo at least one switch between CTMC models in a highly asymmetric manner (see Fig. 1). The two CTMC models are also characterized by pronounced differences in transition/transversion ratios. In conclusion, we show that appropriately modeling compositional heterogeneity for these eubacterial sequences enables inference of the correct phylogeny as well as base frequency compositions that reflect the presence of both mesophilic and thermophilic sequences in the data set.

### Plant Plastid Genes

**Figure 2:**
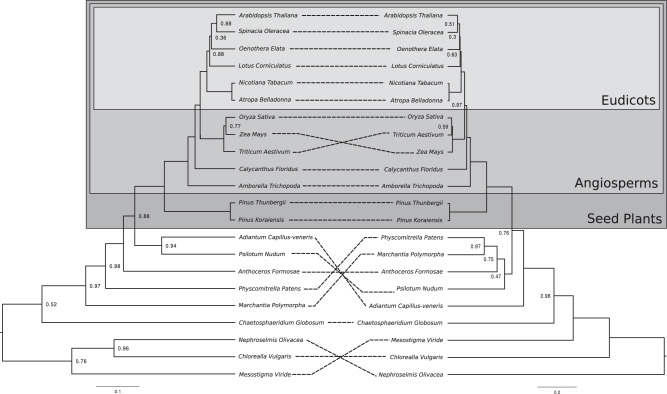
Phylogenetic reconstruction of plant plastid sequences, based on the *psaB * protein-coding gene; unlabeled nodes have $>$0.9999 posterior probability. Left: MCC tree based on a standard GTR+ASRV model. Right: MCC tree based on an MMM(GTR)$_{33A}$ with ASRV, which is strongly supported over the MCC tree generated under the GTR model (log Bayes factor of 347). While only a single different clustering can be observed within the *Angiosperms*, many differing clusters that have very high posterior probabilities are generated using the MMM(GTR)$_{33A}$ outside of the seed plants.

We consider nucleotide sequence data from the protein-coding genes of 23 completely sequenced plant plastid genomes, previously analyzed by Ané et al. (2005) to measure the independence of the substitution process between two groups of taxa as a means of detecting covarion evolution. Assuming a fixed underlying reference tree that represents the likely relationships of plant taxa for which complete chloroplast sequences were available at the time, the covarion test of Ané et al. (2005) detected significant covarion evolution ($P < 0.0005$) in 14 of 57 genes analyzed across all positions. We here analyze the *psaB* gene with standard nucleotide substitution models and MMMs and compare the inferred phylogenies and model fit; we refer to Supplementary material available on Dryad for our analysis of the *ndhD* gene.

A comparison of standard nucleotide substitution models reveals that the combination of a GTR model and an ASRV model, along with a relaxed clock assuming an underlying lognormal distribution, yields the highest (log) marginal likelihood for both data sets. We conduct analyses with MMMs that feature an HKY or GTR substitution model with a single set of symmetric rate parameters along with two or three different sets of base frequencies (i.e., MMM($*$)$_{12*}$ and MMM($*$)$_{13*}$ models), as well as generalizations of these MMMs that feature as many different sets of rate parameters as sets of base frequencies, and both symmetric and asymmetric $\mathbf{\Phi}$ (i.e., MMM($*$)$_{22*}$ and MMM($*$)$_{33*}$ models). For all of these models, we set all $\rho_{i}$ in equation 1.2 to 1 to ensure identifiability when using an ASRV model in combination with MMMs. We also analyze the data with a nucleotide covarion model (Tuffley and Steel 1998), which we can easily compose within our MMM framework through XML specification.

The *psaB* data set strongly prefers the covarion-style model over a standard GTR+ASRV substitution model by a log Bayes factor of 208. The MMM(GTR)$_{12A}$ and MMM(GTR)$_{22A}$ yield log Bayes factors of 257 and 313, respectively, over the standard GTR+ASRV model. MMM(GTR)$_{13A}$ and MMM(GTR)$_{33A}$ parameterizations yield further increases in model fit of 321 and 347, respectively, over the GTR+ASRV model. Because additional categories within the MMM offer diminishing returns in terms of model fit at the expense of additional computation time, we did not explore MMMs with even higher dimensions. Figure 2 shows the maximum clade credibility (MCC) trees obtained under the standard GTR+ASRV model and the MMM(GTR)$_{33A}$ that generated the highest (log) marginal likelihood. While the clustering within the seed plants is identical under both models, substantial differences in posterior support can be observed for specific clades. In the remaining part of the tree, these models result in completely different clustering patterns with strong support for many clades under the MMM(GTR)$_{33A}$ model.

**Figure 3:**
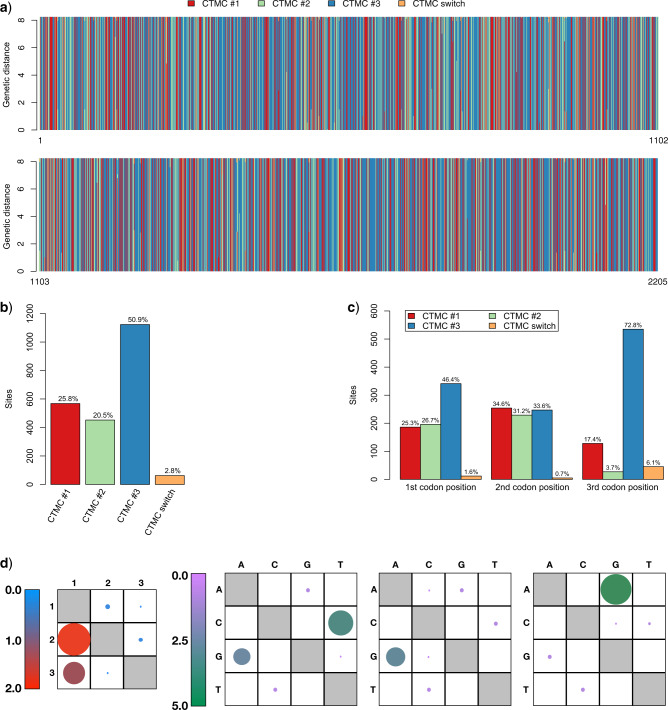
Markov-modulated model behavior on the *psaB * protein-coding gene phylogeny. a) Amount of time (branch lengths in genetic distance) spent in each CTMC model for each alignment site based on the most probable hidden state realizations of the MMM on the maximum a posteriori phylogeny. b) Summary of the number of sites that evolve according to each CTMC, illustrating complex substitution patterns that go beyond codon position partitioning, as well as 2.8% of sites switching between CTMC realizations. c) Distribution of sites in each codon position across the different CTMC model realizations, showing that first and second codon positions switch far less frequently between CTMC models than the third codon position, in which the substitutions occur according to a clearly predominant CTMC. d) Switching behavior of the MMM between the three CTMC models, with the mean instantaneous substitution rates shown for those models (with circle sizes proportional to rate intensity).

In Figure 3, we illustrate the complex substitution patterns across all sites on the MAP *psaB * phylogeny, using the most probable hidden state realizations of the MMM(GTR)$_{33A}$. We use a simple counting procedure to quantify the number of differences between the ancestral model states as a means to reconstruct which sites evolve according to which CTMC within the MMM(GTR)$_{33A}$, and we observe a relatively small amount of CTMC switching throughout the phylogeny (of note, we observe a 4.5-fold increase in number of sites switching between CTMCs in our analysis of the *ndhD * gene in Supplementary material available on Dryad). The reconstructed patterns go beyond mere codon position partitioning, as we observe different substitution dynamics per codon position. In particular, the third codon position is the only position that evolves according to a particular CTMC a majority of the time, and it also exhibits the greatest degree of switching between CTMC realizations. We depict the mean posterior instantaneous substitution rates of the various MMM components in Figure 3, showing a clearly asymmetric CTMC switching process and three distinct GTR model realizations within the MMM. This complex interplay of model components is consistent with the strong Bayes factor support of the MMM(GTR)$_{33A}$ over all other models tested.

## Conclusion

MMMs can infer substantially different phylogenies compared to standard nucleotide substitution models, and they can be associated with significant increases in model fit. A targeted simulation study that assesses the ability of MMMs to retrieve the generative models of simulated sequence alignments and to quantify their increase in model fit when the MMM was the generative model shows that these large differences are not artifacts of using such high-dimensional models (see Supplementary material available on Dryad). Our simulation study also shows similar differences in model fit compared to the ones obtained in this section for the *psaB* and *ndhD* genes, as well as the ability of state-of-the-art Bayesian model selection to select the generative substitution model even when compared with similar model parameterizations. Importantly, when simulating data under a standard GTR model, MMMs exhibit a worse model fit than under the generative GTR model. These analyses of simulated data show that MMMs can easily be used in combination with recent developments in Bayesian model selection (Baele et al. 2016) and provide additional support for our conclusions that these models can yield substantial increases in model fit over standard nucleotide substitution models.

We note that each additional CTMC within an MMM (significantly) increases computational demands, and that a search for the optimal MMM may therefore prove time-consuming for complex large data sets. Avoiding direct evaluation of the finite-time transition probabilities through emerging algorithms that instead manipulate the matrix exponential action (Ji et al. 2016) represents a possible work around. In the mean time, to make such computations manageable, BEAST can however exploit the BEAGLE library (Ayres et al. 2019) to offload the large matrix multiplications onto powerful multi-core hardware solutions. In particular, the use of graphics cards for scientific computing yields significant performance gains over standard multi-core processors (see Supplementary material available on Dryad), rendering phylogenetic inference under these MMMs feasible despite their complexity.

Finally, it remains important to recognize that phylogenetic substitution models draw inspiration from biology and biochemistry, but do not capture the full complexity of these underlying processes. MMMs offer a substantial increase in model complexity over traditional substitution models but—like most other substitution models—also make simplifying assumptions, for example, regarding site-independent evolution, as there is no mechanism within an MMM in which changes in one site result in concomitant changes in another. Resulting model misspecification (and potential overparameterization) can mislead model-based tree reconstruction methods (Steel 2005). To guard against such situations, a well-developed statistical theory such as Bayesian model testing should be employed to compare models in an objective manner and choose a model that carefully balances the model’s parameterization with the available information in the data. After all, as Steel (2005) sagely states, the aim of model selection is not to find the “true model” but to find a model with sufficient parameters to capture the key features of the data.

Additionally, we have made available an online tutorial on how to construct XML files to perform phylogenetic inference using Markov-modulated models in BEAST: http://beast.community/markov_modulated.html.

## Supplementary Material

Data available from the Dryad Digital Repository: https://doi.org/10.5061/dryad.230s5h0.

## References

[B1] Ané C., Burleigh J.G., McMahon M.M., Sanderson. M.J. 2005 Covarion structure in plastid genome evolution: a new statistical test. Mol. Biol. Evol. 22:914–924. 15625184 10.1093/molbev/msi076

[B2] Ayres D. L., Cummings M. P., Baele G., Darling A.E., Lewis P.O., Swofford D.L., Huelsenbeck J.P., Lemey P., Rambaut A., Suchard M.A. 2019 BEAGLE 3: improved performance, scaling, and usability for a high-performance computing library for statistical phylogenetics. Syst. Biol. 68:1052–1061. 31034053 10.1093/sysbio/syz020 PMC6802572

[B3] Baele G., Lemey P., Suchard M.A. 2016 Genealogical working distributions for Bayesian model testing with phylogenetic uncertainty. Syst. Biol. 65:250–264. 26526428 10.1093/sysbio/syv083 PMC5009437

[B4] Blanquart S., Lartillot N. 2006 A Bayesian compound stochastic process for modeling nonstationary and nonhomogeneous sequence evolution. Mol. Biol. Evol. 23:2058—2071. 16931538 10.1093/molbev/msl091

[B5] Embley T.M., Thomas R.H., Williams R.A.D. 1993 Reduced thermophilic bias in the 16s rDNA sequence from *Thermus ruber* provides further support for a relationship between Thermus and Deinococcus. Syst. Appl. Microbial. 16:25–29.

[B6] Felsenstein J. 1981 Evolutionary trees from DNA sequences: a maximum likelihood approach. J. Mol. Evol. 17:368–376. 7288891 10.1007/BF01734359

[B7] Fischer W., Meier-Hellstern K. 1993 The Markov-modulated Poisson process (MMPP) cookbook. Perform. Evaluation 18:149–171.

[B8] Gascuel O., Guindon S. 2007 Modelling the variability of evolutionary processes. Reconstruct. Evol. 2:65–99.

[B9] Guindon S., Rodrigo A.G., Dyer K.A., Huelsenbeck J.P. 2004 Modeling the site-specific variation of selection patterns along lineages. Proc. Natl. Acad. Sci. USA 101:12957–12962. 15326304 10.1073/pnas.0402177101 PMC516501

[B10] Ji X., Griffing A., Thorne J.L. 2016 A phylogenetic approach finds abundant interlocus gene conversion in yeast. Mol. Biol. Evol. 33:2469–2476. 27297467 10.1093/molbev/msw114 PMC6398807

[B11] Mooers A.O., Holmes E.C. 2000 The evolution of base composition and phylogenetic inference. Trends Ecol. Evol. 15:365–369. 10931668 10.1016/s0169-5347(00)01934-0

[B12] Murray R.G.E. 1992 The family Deinococcaceae In: Balows, A., Trüper, H.G., Dworkin, M., Harder, W., and Schleifer, K.-H., editors. The prokaryotes: a handbook on the biology of bacteria: ecophysiology, isolation, identification, applications, Vol. 4. New York: Springer p. 3732—3744.

[B13] Pagel M., Meade A. 2004 A phylogenetic mixture model for detecting pattern-heterogeneity in gene sequence or character-state data. Syst. Biol. 53:571–581. 15371247 10.1080/10635150490468675

[B14] Pan V.Y., Chen Z.Q. 1999 The complexity of the matrix eigenproblem. Proceedings of the Thirty-first Annual ACM Symposium on Theory of Computing STOC ’99 ACM, New York, NY, USA. p. 507–516.

[B15] Penny D., McComish B.J., Charleston M.A., Hendy M.D. 2001 Mathematical elegance with biochemical realism: the covarion model of molecular evolution. J. Mol. Evol. 53:711–753. 11677631 10.1007/s002390010258

[B16] Singer C.E., Ames B.N. 1970 Sunlight ultraviolet and bacterial DNA base ratios. Science 170:822–826. 5473414 10.1126/science.170.3960.822

[B17] Steel M. 2005 Should phylogenetic models be trying to ‘fit an elephant’? Trends Genet. 21:307–309. 15922824 10.1016/j.tig.2005.04.001

[B18] Suchard M.A., Lemey P., Baele G., Ayres D.L., Drummond A.J., Rambaut A. 2018 Bayesian phylogenetic and phylodynamic data integration using BEAST 1.10. Virus Evol. 4:vey016. 29942656 10.1093/ve/vey016 PMC6007674

[B19] Suchard M.A., Rambaut A. 2009 Many-core algorithms for statistical phylogenetics. Bioinformatics 25:1370–1376. 19369496 10.1093/bioinformatics/btp244 PMC2682525

[B20] Tuffley C., Steel M. 1998 Modeling the covarion hypothesis of nucleotide substitution. Math. Biosci. 147:63–91. 9401352 10.1016/s0025-5564(97)00081-3

[B21] Yang Z. 1994 Maximum likelihood phylogenetic estimation from DNA sequences with variable rates over sites: approximate methods. J. Mol. Evol. 39:306–314. 7932792 10.1007/BF00160154

[B22] Yang Z. 1996 Among-site rate variation and its impact on phylogenetic analyses. Trends Ecol. Evol. 11:367–372. 21237881 10.1016/0169-5347(96)10041-0

